# Retraction Note: Fermitin family member 2 promotes melanoma progression by enhancing the binding of p-α-Pix to Rac1 to activate the MAPK pathway

**DOI:** 10.1038/s41388-025-03524-8

**Published:** 2025-08-05

**Authors:** Shaobin Huang, Wuguo Deng, Peng Wang, Yue Yan, Chuanbo Xie, Xiaoling Cao, Miao Chen, Changlin Zhang, Dingbo Shi, Yunxian Dong, Pu Cheng, Hailin Xu, Wenkai Zhu, Zhicheng Hu, Bing Tang, Jiayuan Zhu

**Affiliations:** 1 The First Affiliated Hospital of Sun Yat-sen University, Guangzhou, China; 2 Sun Yat-sen University Cancer Center, State Key Laboratory of Oncology in South China, Collaborative Innovation Center of Cancer Medicine, Guangzhou, China; 3 The Sixth Affiliated Hospital, Sun Yat-sen University, Guangzhou, China; 4 Department of Chemistry, Portland State University, Portland, OR USA

Retraction to: *Oncogene* 10.1038/s41388-021-01954-8, published online 28 July 2021

The Editors-in-Chief have retracted this article. After publication, a number of research integrity related concerns were raised, including:

- Figure 2B appears to contain two overlapping panels.
- a panel in Figure 5G appears to overlap with a panel in 2C with frameshift.
- two panels in Figure 2D MeWo appear to partially overlap.
- two panels in Figure 2C A375 appear to overlap.

The authors were also unable to provide sufficient ethics approval documentation for their study upon request. The Editors have lost confidence in the data and conclusions of this article.

Huang Shaobin has stated on behalf of Hu Zhicheng and Zhu Jiayuan that they agree with this retraction. All other authors have not responded to correspondence from the publisher regarding this retraction.

